# Evaluating pancreatitis in primary care: a population-based cohort study

**DOI:** 10.3399/bjgp14X679732

**Published:** 2014-04-28

**Authors:** Nisha Hazra, Martin Gulliford

**Affiliations:** Department of Public Health Sciences and NIHR Biomedical Research Centre at Guy’s and St Thomas’ Hospitals, London.; Department of Public Health Sciences and NIHR Biomedical Research Centre at Guy’s and St Thomas’ Hospitals, London.

**Keywords:** alcohol abuse, gallstones, incidence, mortality, pancreatitis, primary care

## Abstract

**Background:**

Pancreatitis is an important condition with significant mortality. Primary care may have an important role to play in its prevention, early diagnosis, and ongoing management.

**Aim:**

To evaluate incidence, case fatality, and clinical features of acute and chronic pancreatitis in a large population.

**Design and setting:**

Population-based cohort study using a primary care database in the UK from 1990 to 2013.

**Method:**

Use of general practice records from 16 491 patients diagnosed with pancreatitis. Age-standardised incidence rates and case fatality were estimated. Clinical features, aetiology, and patterns of recurrence were evaluated.

**Results:**

Incidence of pancreatitis increased from 14.8 in 100 000 (1990–1994) to 31.2 in 100 000 (2010–2013) in males, and from 14.5 to 28.3 in 100 000 in females (2010–2013). Overall case fatality after diagnosis was 4.3% (95% CI = 4.0% to 4.6%) at 90 days and 7.9% (95% CI = 7.5% to 8.4%) at 365 days. In 1990–1994, 10% of patients with acute pancreatitis were recorded as heavy drinkers, increasing to 12% in 2010–2012; for patients with chronic pancreatitis the proportions were 13%, rising to 21%. Among patients who died in the 90 days after diagnosis, 92% consulted with their general practice in the 2 months before first diagnosis.

**Conclusion:**

The incidence of pancreatitis is increasing over time. Alcohol abuse may now account for at least one in eight cases of acute, and one in five cases of chronic pancreatitis. Consultations among those who subsequently died may have offered potential for earlier diagnosis and intervention.

## INTRODUCTION

Pancreatitis is characterised by an inflammatory process involving the pancreas with consequent damage to its structure and function.^1^ Acute pancreatitis generally has a sudden onset and carries a significant case fatality, whereas chronic pancreatitis may reoccur over many years leading to malnutrition and chronic ill health. The mortality rate of pancreatitis is substantial but estimates vary considerably across studies, between 4% and 15% for all cases and between 15% and 90% for more severe cases.^2^,^3^ Mortality has fallen steadily over the past 30 years, and this reduction has coincided with the recognition and application of indices of severity of illness and improved intensive care support.^3^,^4^

Recent reports have shown an increase in the incidence of pancreatitis.^5^–^9^ This increase emphasises the importance of a public health perspective on the condition and the relevance of strategies for primary prevention, early diagnosis, and prevention of recurrence. However, there have been few population-based studies of pancreatitis and most of these have analysed data from hospital admissions as the primary information source.^6^–^9^ The present study aimed at evaluating the epidemiology and clinical features of pancreatitis using electronic health records from a large and representative sample of family practices in the UK from 1990 to 2013. This approach is designed to capitalise on the registration of more than 98% of the UK population with NHS family practices,^10^ with the maintenance of longitudinal electronic records which may span many years of patient follow-up. This offers the opportunity to explore antecedent events before diagnosis and aetiological factors recorded over time, as well as longer-term outcomes of mortality and recurrence. The present study, therefore, aimed at estimating trends in the incidence of pancreatitis in primary care, mortality from the condition, and clinical features including presenting symptoms, patterns of recurrence, and the changing role of aetiological factors over time.

## METHOD

### Data source

A population-based cohort study was conducted of patients diagnosed with pancreatitis in the Clinical Practice Research Datalink (CPRD) between 1990 and 2013. The CPRD is the world’s largest primary care database, containing well described and valid data^11^,^12^ from several hundred family practices in the UK. Data elements include demographics, prescriptions, clinical events and diagnoses, referrals to hospitals, and additional patient information, such as height, weight, age, alcohol use, immunisations, and deaths. Diagnoses recorded in CPRD have been shown to have high predictive value in validation studies.^11^ For entry into the CPRD, practice data must be up to the standard for research as set out by the CPRD group. Fully anonymised data were used for analysis.

**How this fits in**

Pancreatitis is an important condition with significant mortality and GPs may have an important role to play in its prevention, early diagnosis, and ongoing management. This study evaluated the incidence, clinical features, and mortality rates of pancreatitis from a primary care perspective. The results reveal a rapid increase in the occurrence of pancreatitis, with most general practices observing between two and four new cases a year. One in 13 patients dies within 12 months of first diagnosis and has generally made several visits to their general practice in the 2 months before diagnosis. Alcohol abuse may now account for at least one in eight cases of acute pancreatitis and one in five cases of chronic pancreatitis.

### Study population

The initial study cohort included 19 182 patients registered at general practices providing up-to-standard data to CPRD with a record of pancreatitis between 1 January 1990 and 30 April 2013. Participants had a diagnosis of pancreatitis recorded based on Read Codes recorded in the clinical and referral files. Four Read Codes accounted for approximately 93% of all cases including pancreatitis (J670z11), acute pancreatitis (J670.00 and J670z00), and chronic pancreatitis (J671.00). Patients were classified as ‘chronic’ if they were initially diagnosed with chronic pancreatitis, but as ‘acute’ pancreatitis otherwise. The index date was defined as the date of the first recorded pancreatitis event. The ‘start of record’ for each case was defined as the later of the patient’s registration date at a CPRD practice, or the date the practice joined CPRD and provided up-to-standard data. The ‘end of record’ for each case was defined as the earliest of the death date, the end of registration date, or the last data collection date. Cases were classified as ‘incident’ cases if the patients were diagnosed >1 year after the start of the CPRD record. There were 16 491 incident cases of pancreatitis included in the analysis.

### Statistical analysis

Stata (version 13.0) was used to conduct all statistical analysis. Incidence rates were estimated using incident pancreatitis cases as the numerator and person years for acceptable patients in CPRD population as the denominator. These were calculated by sex and aggregated into 5-year periods. Confidence intervals (CIs) were estimated using the Poisson distribution and age-standardisation was performed using the 2013 European Standard Population^13^ for reference.

All symptoms and clinical features of the cohort were determined by evaluating the frequency distribution of relevant ‘Read Codes’ and ‘Read Terms’ associated with pancreatitis. Symptoms were reported for four time periods: from 60–30 days before diagnosis, from 30 days before to the diagnosis date, from diagnosis to 30 days after, and from 30–60 days after diagnosis. Subdivisions of the Read Code classification were used for indigestion (Read Codes 195*), type of gastrointestinal tract pain (196*), site of gastrointestinal tract pain (197*), nausea (198*), vomiting (199*), abdominal distension (19A*), flatulence (19B*), constipation (19C*), faeces symptoms (19E*), diarrhoea (19F*), diarrhoea and vomiting (19G*), backache symptoms (16C* and N14*), other gastrointestinal symptoms (R09*), and gallstones and their complications (J64* and J65*). Case fatality was calculated at 90 and 365 days after first diagnosis of pancreatitis. Nelson–Aalen cumulative hazard functions and their 95% CIs were determined for patients with acute pancreatitis, patients with chronic pancreatitis, and overall. The number of consultations and referrals in the 60 days before diagnosis were evaluated in relation to mortality after diagnosis, allowing a maximum of one event a day.

The two main aetiologies of pancreatitis, gallstones and alcohol, were evaluated by type of pancreatitis. Drinking status was determined using Read Codes related to drinking behaviour, as well as additional information on alcohol consumption recorded in CPRD. Males drinking more than 21 units a week, and females drinking more than 14, were classified as heavy drinkers. The drinking status closest to diagnosis was used to classify patients as non-drinkers, moderate drinkers, heavy drinkers, or ex-drinkers. Patients were considered to have a gallstone aetiology if they ever had Codes for gallstones or gallbladder disease recorded (J64* and J65*). Aetiological status was described by period of diagnosis and aggregated into 5-year periods. A recurrence was defined as a record of pancreatitis after a period of 60 days free from a pancreatitis Code. Patterns of recurrence were described using the number of attacks after first pancreatitis event, for acute cases, chronic cases, and overall. Amylase test results were also identified where available in CPRD during the 60 days before and after diagnosis. Maximum values for each patient during each time period were considered.

## RESULTS

The study cohort consisted of 16 491 incident cases (8121 male, 8370 female), with 14 723 (89%) initially classified as acute and 1768 (11%) as chronic. The proportion of cases diagnosed as chronic increased from 8% in 1990–1994 to 11% in 2010–2013. The median age at diagnosis in males was 58 years (interquartile [IQR] range 44–71 years), and in females was 60 years (IQR 44–74 years). Most general practices observe between two and four new cases of pancreatitis a year.

Age-standardised incidence of pancreatitis increased in males from 14.8 in 100 000 in 1990–1994 to 31.2 in 100 000 in 2010–2013, and in females from 14.5 to 28.3 in 100 000 over the same period (Table 1). The annual increment was approximately 0.84 in 100 000 each year in males (*P*<0.001) and 0.74 in 100 000 each year in females (test for linear trend over time, *P*<0.001).

**Table 1.** Age-standardised incidence rates (95% CI) per 100 000 person-years for pancreatitis in the CPRD population, according to period of diagnosis and sex^a^

**Period**	**Incident cases of pancreatitis**	**Person-years, millions**	**Incidence rate per 100 000 person years (95% CI)**
**All**			
1990–1994	833	6.1	14.65 (12.65 to 16.65)
1995–1999	1825	11.0	17.34 (15.74 to 18.94)
2000–2004	4110	20.5	20.95 (19.66 to 22.23)
2005–2009	5738	23.8	25.08 (23.77 to 26.38)
2010–2013	3980	13.9	29.57 (27.73 to 31.41)

**Males**			
1990–1994	390	3.0	14.83 (13.32 to 16.34)
1995–1999	906	5.4	18.38 (17.16 to 19.60)
2000–2004	1993	10.2	21.57 (20.60 to 22.54)
2005–2009	2837	11.8	26.23 (25.25 to 27.21)
2010–2013	1992	6.9	31.20 (29.82 to 32.59)

**Females**			
1990–1994	443	3.1	14.45 (13.10 to 15.81)
1995–1999	919	5.6	16.39 (15.32 to 17.46)
2000–2004	2117	10.3	20.50 (19.62 to 21.38)
2005–2009	2901	12.0	24.21 (23.32 to 25.10)
2010–2013	1988	7.0	28.26 (27.01 to 29.51)

a European standard population from 2013 used for reference.

The most commonly recorded clinical manifestations overall among the study cohort were gastrointestinal pain, vomiting, and other abdominal symptoms (Table 2). Diagnoses of gallstones and gallbladder disorders were also often recorded before and after diagnosis. A total of 3474 patients died in the cohort, including 1832 males and 1642 females over the course of the study period, from any cause. Table 3 shows case fatality rates and cumulative deaths for all patients, and for patients first diagnosed with acute or chronic pancreatitis, at 90 days and 365 days after first diagnosis. The overall case fatality after diagnosis of pancreatitis was 4.3% (95% CI = 4.0% to 4.6%) at 90 days and 7.9% (95% CI = 7.5% to 8.4%) at 365 days, based on 695 and 1213 deaths, respectively. In the 60 days up to but not including the day of diagnosis, the median number of days with consultations and referrals recorded by the GP was 3 (IQR 2–5). Among patients who did not die, 15% did not consult in the 60 days before diagnosis, compared with 8% for those who died within either the first 7 or first 90 days after diagnosis (Table 4).

**Table 2.** Frequency of different symptoms recorded in different periods before and after first diagnosis in 16 491 patients with pancreatitis

**Symptom [Code]^a^**	**Time from diagnosis, *n* (%)**			
	**60–31 days before diagnosis**	**30 days before, to diagnosis**	**1–30 days after diagnosis**	**31–60 days after diagnosis**
Gastrointestinal tract pain (type and site) [196 and 197]	604 (3.7)	1897 (11.5)	2,526 (15.3)	781 (4.7)
Cholelithiasis and gall bladder disease [J64/J65]	344 (2.1)	1792 (10.9)	636 (3.9)	289 (1.8)
Other abdominal and pelvic symptoms [R09]	254 (1.5)	851 (5.2)	1,169 (7.1)	347 (2.1)
Vomiting [199]	112 (0.7)	275 (1.7)	416 (2.5)	146 (0.9)
Diarrhoea symptoms [19F]	107 (0.7)	165 (1.0)	145 (0.9)	114 (0.7)
Constipation [19C]	113 (0.7)	191 (1.2)	104 (0.6)	73 (0.4)
None	13 142 (79.7)	5991 (36.3)	6556 (39.8)	12 806 (77.7)

a Symptoms less than 1% in all four periods are not presented in table: Indigestion symptoms [195], Nausea [198], Abdominal distension symptom [19A], Flatulence/wind [19B], Tenesmus [19D], Faeces/motions [19E], Diarrhoea and vomiting [19G], Backache symptom [16C], Other and unspecified back disorders [N14].

**Table 3.** Case fatality at 90 days and 1 year from first diagnosis for 16 491 patients with pancreatitis

**Status**	**Number at risk**	**90 days**		**365 days**	
		**Deaths, *n***	**Case fatality,^a^ % (95% CI)**	**Deaths, *n***	**Case fatality, ^a^ % (95% CI)**
All	16 491	695	4.3 (4.0 to 4.6)	1213	7.9 (7.5 to 8.4)
Acute	14 723	627	4.4 (4.0 to 4.7)	1037	7.5 (7.1 to 8.0)
Chronic	1768	68	4.0 (3.1 to 5.0)	176	11.0 (9.5 to 12.8)

a Nelson-Aalen Cumulative Hazard Function.

**Table 4.** Number of consultations or referrals at the practice recorded in the 60 days before diagnosis in relation to mortality after diagnosis

**Number of consultations and referrals within 60 days before diagnosis**	**Died 0–7 days after diagnosis, *n* (%)**	**Died 8–89 days after diagnosis, *n* (%)**	**Died≥90 days after diagnosis, *n* (%)**	**Alive, *n* (%)**
0	33 (8)	23 (8)	366 (13)	1915 (15)
1–2	152 (38)	95 (32)	932 (34)	4346 (33)
3–4	102 (26)	65 (22)	634 (23)	3122 (24)
≥5	112 (28)	111 (38)	849 (31)	3634 (28)
Total	399	294	2781	13 017

Table 5 presents data for the proportion of patients classified as heavy drinkers or with a history of gallstones or gallbladder disease. Overall, the proportion of heavy drinkers increased from 10% to 13% between 1990 and 2013. However, among chronic cases the proportion increased from 13% to 21%. The proportion with gallstones ever recorded declined from 30% to 28% among all cases and from 22% to 15% for chronic cases.

**Table 5.** Aetiological factors for acute and chronic pancreatitis by time period

**Period of diagnosis**	**All, *n* (%)**			**Acute, *n* (%)**			**Chronic, *n* (%)**		
	**Heavy drinker**	**Gallstones^a^**	**Total**	**Heavy drinker**	**Gallstones^a^**	**Total**	**Heavy drinker**	**Gallstones^a^**	**Total**
1990–1994	82 (10)	256 (30)	838 (100)	73 (10)	241 (31)	771 (100)	9 (13)	15 (22)	67 (100)
1995–1999	217 (12)	580 (32)	1825 (100)	190 (12)	541 (33)	1655 (100)	27 (16)	39 (23)	170 (100)
2000–2004	535 (13)	1213 (30)	4110 (100)	433 (12)	1152 (32)	3638 (100)	102 (22)	61 (13)	472 (100)
2005–2009	777 (14)	1659 (29)	5738 (100)	634 (12)	1568 (31)	5106 (100)	143 (23)	91 (14)	632 (100)
2010–2012	527 (13)	1106 (28)	3980 (100)	439 (12)	1044 (29)	3553 (100)	88 (21)	62 (15)	427 (100)
All	2138 (13)	4814 (29)	16 491 (100)	1759 (12)	4546 (31)	14 723 (100)	369 (21)	268 (15)	1768 (100)

a History of gallstones ever.

Table 6 presents data for recurrence of pancreatitis defined as a 60-day period free from pancreatitis Codes. Using this definition, during the study period, 12 156 (74%) experienced only one incident of pancreatitis, 3181 (19%) experienced two, 712 (4%) experienced three, and 442 (2.7%) experienced four or more with similar patterns of recurrence for patients initially diagnosed either as acute or chronic. Amylase test results were only available for 5% of patients during the 60 days before diagnosis and 12% of patients during the 60 days after diagnosis. Serum amylase levels greater than 500 U/l were observed in 1.6% of patients during the 60 days before diagnosis, compared with 3.1% of patients during the 60 days after diagnosis.

**Table 6.** Recurrent attacks^a^ (recorded diagnoses) after first record of pancreatitis

**Number of attacks after first**	**Acute, *n* (%)**	**Chronic, *n* (%)**	**All, *n* (%)**
0	10 826 (74.5)	1330 (75.2)	12 156 (73.7)
1	2936 (19.9)	245 (13.9)	3181 (19.3)
2	607 (4.1)	105 (5.9)	712 (4.3)
3	205 (1.4)	56 (3.2)	261 (1.6)
4	81 (0.6)	18 (1.0)	99 (0.6)
5	34 (0.2)	4 (0.2)	38 (0.2)
6	20 (0.1)	5 (0.3)	25 (0.2)
≥7	14 (0.1)	5 (0.3)	19 (0.1)
*N* (%) patients	14 723 (100)	1768 (100)	16 491 (100)

a A record of pancreatitis after a period of 60 days free from a pancreatitis code.

## DISCUSSION

### Summary

This study provides evidence of the changing epidemiology of pancreatitis in the UK. The age-standardised incidence of pancreatitis has more than doubled over two decades. There has been an increase in the proportion of patients who are recorded as heavy consumers of alcohol, as well as an increase in the proportion of patients with chronic pancreatitis. A continuing burden of morbidity over time in patients diagnosed with pancreatitis is suggested by the recording of recurrent diagnoses, for patients initially diagnosed with acute pancreatitis as well as those initially diagnosed with chronic pancreatitis. The predominant clinical features preceding initial diagnoses include abdominal pain and vomiting. The frequent recording of diagnoses associated with gallstones could suggest that pancreatitis occurs in the context of cholelithiasis, or that the condition is initially diagnosed as biliary colic and related disorders. Patients made a median of three contacts with their general practice before diagnosis, which suggests a potential for earlier diagnosis in some cases. GPs recognise the importance, however, of seeing patients on more than one occasion to reach a diagnosis, particularly when the initial symptoms, as in these cases, are non-specific. Among patients who died soon after diagnosis, only 8% had not consulted their general practice within the 60 days leading up to diagnosis, compared with 15% among those who did not die.

### Strengths and limitations

The study had the strengths of a large sample drawn from a large representative population of UK general practices. Use of primary care electronic records allowed characterisation of the clinical features, and prognosis over time, before and after a diagnosis of pancreatitis. Diagnoses recorded in CPRD generally have a high predictive value,^14^ but direct validation of the diagnoses was not available in these cases. Codes recorded for acute and chronic pancreatitis will be quite robust; however, recording patterns of symptom Codes can vary across practitioners. Serum amylase test results were not widely recorded in CPRD.

Previous studies have used hospital records, for which laboratory confirmation of the diagnosis might be more commonly available. However, Spanier *et al* report that there are concerns around the reliability of reported incidence rates that depend heavily on hospital data.^15^ There may be problems of ascertainment if diagnoses made in hospital are not coded into primary care records. However, there may be cases diagnosed in the community that do not reach hospital. The present study used the registered population as denominator, which might lead to slight underestimation of rates compared with the resident population. Relevant symptoms were not recorded for a high proportion of cases, but the data available should provide reliable evidence for the relative frequency of different symptoms. If symptoms were more consistently recorded, diagnostic delay could be better understood. Recording of alcohol habits may be incomplete and there may have been important trends in recording over time. The present data are likely to underestimate the importance of alcohol as an aetiological factor for pancreatitis. Read Codes for heavy drinking will be quite robust; however, patients classified as heavy drinkers based on quantity consumed will be underestimated as patients tend to underreport.

### Comparison with existing literature

Previous studies on the incidence and case fatality of pancreatitis in England,^6^ Wales,^7^ Netherlands,^8^ and Denmark^9^ have used hospital data to report on incidence and case fatality for pancreatitis. The English study used national record linkage of inpatient and mortality data for 52 096 patients between 1998 and 2003 to describe trends in incidence, case fatality, and geographical variation. The calculated overall incidence was 22.4 in 100 000 population, increasing by 3.1% annually. Case fatality was 6.7% at 60 days, and was higher for heavy drinkers compared with those with a history of gallstones. This is consistent with the present results, but the present data are the first to demonstrate a continuing increase in mortality up to 1 year after the diagnosis of pancreatitis. Roberts’^7^ data for cholelithiasis aetiology is similar to the present data, but the study reported a higher frequency of alcoholic aetiology, particularly among young males living in Wales. The Dutch study used hospital data to analyse trends in incidence and mortality for 12 million patients between 1995 and 2005. The calculated overall incidence was 14.7 in 100 000 population in 2005, with yearly increases of 1.6% for males and 1.9% for females. Although slightly lower than the present results, incidence rates in the Netherlands have previously been reported to be far lower compared with rates from several Scandinavian countries and the US.^15^

The Danish study used the Hospital Discharge Registry for the period 1981–2000 to report sex- and age- standardised incidence rates and 30-day case fatality rates for 2350 incident cases. The calculated incidence rates were 37.8 in 100 000 person years in 2000 for females, and 27.1 in 100 000 person years in 2000 for males. This is similar to the present results however, these showed a greater increase over time in females compared with males. In the present study, incidence increased slightly faster in males compared with females. The overall 30-day case fatality rate was 7.5%, increasing with age and decreasing with time. A limitation of this study is the small sample size. All four of these hospital-based studies did not have sufficient data concerning aetiology of pancreatitis. Roberts *et al* reported aetiology in 34% of the study cohort.^6^ The present study is the first to report aetiology in greater than 50% of the study cohort.

### Implications for research and practice

Large population-based epidemiological studies reporting trends in incidence and mortality for pancreatitis in primary care are scarce. This study introduces new information regarding the clinical features and aetiological factors of patients with pancreatitis in general practice. There exists potential for future research in this area, as well as around patterns of therapy for managing the disease. There is also potential for research into the increased risk of pancreatitis during cholecystectomy. This research will have implications for clinical practice, as well as potential for earlier diagnosis of pancreatitis in primary care. Results also identify opportunities for prevention, since the proportion of cases attributable to alcohol abuse may be increasing.

## Open access

This article is Open Access: CC BY NC 3.0 license (http://creativecommons.org/licenses/by-nc/3.0/).
